# Role of beta-blockers in Marfan's syndrome and bicuspid aortic valve: A time for re-appraisal

**DOI:** 10.4103/0974-2069.43885

**Published:** 2008

**Authors:** Balu Vaidyanathan

**Affiliations:** Department of Pediatric Cardiology, Amrita Institute of Medical Sciences, Kochi, India

**Keywords:** Marfan's syndrome, aortic root dilatation, aneurysm

## INTRODUCTION

Marfan's syndrome is an autosomal dominant disorder with high penetrance having an incidence of 1 in 5000 live births.[1] It has heterogenous clinical presentation and the diagnosis is based on the Ghent's criteria. The recognized clinical features include tall stature, arachnodactyly, joint hypermobility, skeletal abnormalities, lens subluxation, and mitral valve prolapse. The genetic basis for Marfan's syndrome is a mutation in the *fibrillin-1* gene, and till date about 600 different types of mutations in this gene have been reported.[1]

The major cause of morbidity and mortality in patients with Marfan's syndrome is related to aortic dilatation leading to aortic dissection or rupture and aortic valve regurgitation. This condition worsens with age and at the age of 30 years, men and women with Marfan's syndrome have an annual death risk of 2% and 1%, respectively – 20–40 times higher than normal population of the same age.[2] Seventy percent of the deaths in Marfan's syndrome can be directly attributed to acute cardiovascular complications, especially aortic dissection.[2] Hence, the most important target for improving survival in patients with Marfan's syndrome is to prevent or delay aortic dissection.

A review of clinical studies of medical treatment for Marfan's syndrome reveals that only three classes of drugs have been investigated – beta-blockers, angiotensin converting enzyme (ACE) inhibitors, and calcium channel blockers with primary focus being on beta-blockers.[3] The proposed mechanisms of benefit of beta-blockers in Marfan's syndrome include reduction in the rate of pressure increase in aorta (d*P/*d*t*) and reduction in heart rate, which reduces the number of systolic impulses in a given period.[4] To date, randomized control studies supporting this management approach has been limited, especially in children. The lacunae in our current knowledge about beta-blocker therapy in Marfan's syndrome include effects on reducing rate of aortic root dilatation, impact on clinical outcomes, and the timing to initiate therapy in patients who may have potential benefit with such therapy. The clinician will need to consider the potential side effects of such a long-term therapy in children, especially when the data supporting such treatment is not very robust.

Bicuspid aortic valve is perhaps the most common congenital heart disease occurring at a frequency of 1–2/1000 live births.[5] Several studies have shown the presence of an intrinsic aortic wall pathology in patients with bicuspid aortic valve characterized by abnormal distensibility and pulse wave velocity.[6,7] This impairs elasticity of the vessel wall leading to aortic dilatation. Studies have shown that Marfan's syndrome and bicuspid aortic valve have many histological and clinical similarities with respect to aortic dilatation and aortic complications like dissection.[5] Though, the effect of beta-blockers have been extensively studied in patients with Marfan's syndrome, there is no data regarding the use of beta-blockers in preventing aortic dilatation in patients with bicuspid aortic valve. Extending the data from Marfan's syndrome and considering the similarities in their pathology, it is intuitive to think that there could be potential benefit with such therapy.

This meta-analysis and e-mail survey of expert opinion attempts to clarify the issues involved in medical management of patients with Marfan's syndrome and bicuspid aortic valve with beta-blockers including effects on slowing aortic root dilatation and cardiovascular effects – death, aortic dissection, and surgery – and when to initiate therapy in patients who may potentially benefit from it.

## REVIEW OF CURRENT LITERATURE

### Role of beta-blockers in patients with Marfan's syndrome and bicuspid aortic valve

The first report describing a beneficial effect of beta-blockers in patients with Marfan's syndrome was published by Halpern *et al*., from Johns Hopkins university.[8] This small study in six adult patients showed that beta-blockers could slow the rate of aortic root dilatation in Marfan's syndrome through their negative inotropic and chronotropic effects. The landmark study showing the beneficial effect of beta-blockers on slowing the rate of aortic root dilatation was published by Shores *et al*., in 1994.[9] This open-label study conducted in 70 patients (adult and pediatric) reported lower incidence of clinical endpoints in patients treated with beta-blockers versus controls (16% vs. 24%). A recent study in children with Marfan's syndrome also reported decrease in the rate of aortic dilatation by a mean of 0.16 mm/year in patients treated with beta-blockers versus controls.[10] This study also reported a trend toward lower cardiac mortality, decreased need for preventive aortic surgery, and fewer dissections. However, a study by Tierney *et al*., in children reported that beta-blocker therapy does not significantly alter the rate of aortic root dilatation in children with Marfan's syndrome.[11] A recent meta-analysis also questioned the role of beta-blockers in the medical therapy of patients with Marfan's syndrome.[12] There is no data regarding the use of beta-blockers in patients with bicuspid aortic valve and aortic dilation.

### Effect of beta-blockade on rate of aortic root dilatation and clinical endpoints

The randomized control study conducted by Shores *et al*., showed that the rate of enlargement of aorta in patients receiving treatment was less than one-third the rate of patients receiving no treatment (*P* < 0.001).[9] Also, clinical endpoints were fewer in treated patients (16% vs. 24%), and the study was underpowered to detect effect on mortality. In the study by Ladouceur *et al*.,[10] though the baseline aortic diameters were higher in patients treated with beta-blockers, there was significant slowing of the rate of aortic dilatation (0.16 mm/year) after commencement of beta-blocker therapy. More children required aortic surgery in the control group (6.4% vs. 2.6%), and of the four total deaths, three occurred in the control group. However, Tierney *et al*., reported no significant change in progression of aortic root dilatation between patients treated with beta-blockers versus controls, despite the fact that heart rate was significantly slower in those treated with beta-blockers.[11] A recent meta-analysis also concluded that there is no beneficial effect of beta-blocker therapy on aortic dilatation or clinical events in children with Marfan's syndrome.[12] The differential effects found in the various studies are probably related to variations in study design, the time of onset of therapy (diagnosis vs. onset of aortic dilatation), and different age groups studied.

### When should beta-blocker therapy be initiated?

Most studies had left this decision to the discretion of the treating physician. Most of these decisions were made empirically. There is no clear consensus on whether treatment should be initiated as soon as the diagnosis of Marfan's syndrome is made or one can wait till significant aortic root dilatation has set in. No clear cut-off values of aortic root measurements have been suggested as an indication for starting the therapy. In one study, a greater proportion of treated patients had positive family history of Marfan's syndrome.[11] Some of these factors may have played a role in influencing the rather contradictory results of some of these trials.

### Choice of beta-blocker and monitoring of therapy

Atenolol was the most commonly used agent in most studies.[10–12] In most of the adult studies, the dose of beta-blocker was adjusted based on heart rate response to exercise with a target heart rate of less than 100 beats/minute after exercise.[9] In most of the studies involving children , exercise testing was not performed and the dose of beta-blocker was empirically increased till the target dose was achieved or side-effects encountered. In children, the starting dose was 12.5–25 mg and the target dose was 25 mg, while in adolescents the target dose was 50 mg.[11]

### What are the alternative therapeutic drugs for Marfan's syndrome?

#### Calcium channel blockers

These drugs are sometimes prescribed to patients in whom beta-blockers are contraindicated. In a small study, Rossi-Foulkes *et al*., reported a slower rate of enlargement of the aorta in patients receiving treatment compared with placebo.[13]

#### Angiotensin converting enzyme inhibitors

The potential benefit of these drugs in Marfan's syndrome maybe mediated through angiotensin-II type-2 receptor blockade, thereby, reducing apoptosis of vascular smooth muscle cells and cystic medial necrosis. A recent randomized controlled trial in children reported increased aortic distensibility and reduced stiffness index in patients treated with enalapril compared with beta-blockers.[14]

#### Angiotensin-II type-1 receptor blockers (AT1R blockers)

The postulated mechanism of benefit of these agents is mediated through their transforming growth factor (TGF)-beta receptor antagonism.[15] In a preliminary report, Habashi *et al*., demonstrated that losartan therapy in the mouse model of Marfan's syndrome resulted in complete correction of the phenotypic abnormalities in the aortic wall.[16] These results have prompted a multicenter randomized clinical trial, coordinated by the Pediatric Heart Network of the National Heart, Lung and Blood institute of the National Institutes of Health, comparing losartan with beta-blocker therapy in children and young adults with Marfan's syndrome.

#### Other drugs

Other drugs with theoretical rationale in patients with Marfan's syndrome include matrix metalloproteinase inhibitors (tetracycline, doxycycline), advanced glycation end-product (AGE) receptor blockers, aldosterone receptor antagonists, and drugs to reduce homocystenemia.[3]

### E-mail survey and expert opinion

The e-mail survey was carried out using the following questionnaire. All the experts were requested to respond to the the following questions and a summary of the consensus is presented.

#### Do you think there is any role of beta-blockers in the medical therapy of patients with Marfan's syndrome and bicuspid aortic valve?

*Survey opinion*: Most of the respondents felt that beta-blockers are useful in patients with Marfan's syndrome. Regarding bicuspid aortic valve, most were of opinion that beta-blockers are not of much value, especially if there is no aortic root dilatation. Dr Wilson and Dr Saxena felt that beta-blockers may have a role in bicuspid aortic valve if there is aortic root dilatation. Dr Teirney and Dr Shrivatsava, however, were of opinion that beta-blockers are not effective in both Marfan's syndrome and bicuspid aortic valve.

#### What in your opinion is the effect of beta-blockers in patients with Marfan's syndrome and bicuspid aortic valve as regards:

- Reducing the rate of progression of aortic root dilatation.
- Delaying need for aortic root surgery.
- Reducing mortality and cardiovascular events.

*Survey opinion:* Most of the respondents were of opinion that beta-blockers were useful for all the three endpoints in patients with Marfan's syndrome.

#### If you think treatment with beta-blockers is beneficial, when would you recommend initiation of therapy?

- At time of diagnosis itself.
- Once aortic root dilatation has set in.
- Any cut-off value for aortic root dimension.

*Survey opinion:* Most of the experts preferred to start beta-blockers only after aortic root dilatation has set in. None offered any cut-off values for aortic root dilatation in children. Prof. Jondeau, however, was of opinion that beta-blockers should be started right from the time of diagnosis itself.

#### What is your beta-blocker of choice and how do you monitor adequacy of therapy?

*Survey opinion:* The overall opinion was that the choice of beta-blocker really does not matter, though most respondents preferred cardioselective beta-blockers like atenolol. Atenolol was recommended in standard tolerated doses (1–2 mg/kg/day). Most of the experts recommended monitoring of resting heart rate and tolerance before increasing the dose. Yearly monitoring of the aortic root dimension by echo was also recommended.

#### Do you use any other alternative drugs for medical management for patients with Marfan's syndrome or bicuspid aortic valve with aortic root dilatation?

*Survey opinion:* Most of the experts were not in favor of alternative drugs till more evidence is available. Dr. Wilson suggested use of ACE inhibitors in patients who cannot tolerate beta-blockers and use of calcium channel blockers in those who are intolerant to both beta-blockers and ACE inhibitors. Most of the experts felt that losartan is still in a trial phase for the medical treatment of patients with Marfan's syndrome.

## CONCLUSIONS

It will be a reasonable practice to recommend beta-blockers in patients with Marfan's syndrome with aortic root dilatation, though there is some recent evidence suggesting that such therapy may not be beneficial. Based on available data, beta-blockers may be of value in delaying the progression of aortic root dilatation, while harder clinical endpoints like mortality and vascular complications may not be altered much. The role of newer drugs like losartan in Marfan's syndrome needs further evaluation. In patients with bicuspid aortic valve, there is no evidence base to support the use of any of these drugs at present.
